# Activation of the CREB/*c-Fos* Pathway during Long-Term Synaptic Plasticity in the Cerebellum Granular Layer

**DOI:** 10.3389/fncel.2017.00184

**Published:** 2017-06-28

**Authors:** Daniela Gandolfi, Silvia Cerri, Jonathan Mapelli, Mariarosa Polimeni, Simona Tritto, Marie-Therese Fuzzati-Armentero, Albertino Bigiani, Fabio Blandini, Lisa Mapelli, Egidio D’Angelo

**Affiliations:** ^1^Neurophysiology Unit, Department of Brain and Behavioral Sciences, University of PaviaPavia, Italy; ^2^Brain Connectivity Center, Fondazione Istituto Neurologico Nazionale Casimiro Mondino (IRCCS)Pavia, Italy; ^3^Laboratory of Functional Neurochemistry, Center for Research in Neurodegenerative Diseases, Fondazione Istituto Neurologico Nazionale Casimiro Mondino (IRCCS)Pavia, Italy; ^4^Department of Biomedical, Metabolic and Neural Sciences, Center for Neuroscience and Neurotechnology, University of Modena and Reggio EmiliaModena, Italy; ^5^Department of Public Health, Experimental and Forensic Medicine, Human Anatomy Unit, University of PaviaPavia Italy; ^6^Museo Storico Della Fisica e Centro Studi e Ricerche Enrico FermiRome, Italy

**Keywords:** long-term plasticity, gene expression, CREB, NMDARs, cerebellum

## Abstract

The induction of long-term potentiation and depression (LTP and LTD) is thought to trigger gene expression and protein synthesis, leading to consolidation of synaptic and neuronal changes. However, while LTP and LTD have been proposed to play important roles for sensori-motor learning in the cerebellum granular layer, their association with these mechanisms remained unclear. Here, we have investigated phosphorylation of the cAMP-responsive element binding protein (CREB) and activation of the immediate early gene *c-Fos* pathway following the induction of synaptic plasticity by theta-burst stimulation (TBS) in acute cerebellar slices. LTP and LTD were localized using voltage-sensitive dye imaging (VSDi). At two time points following TBS (15 min and 120 min), corresponding to the early and late phases of plasticity, slices were fixed and processed to evaluate CREB phosphorylation (P-CREB) and c-FOS protein levels, as well as *Creb* and *c-Fos* mRNA expression. High levels of P-CREB and *Creb/c-Fos* were detected before those of c-FOS, as expected if CREB phosphorylation triggered gene expression followed by protein synthesis. No differences between control slices and slices stimulated with TBS were observed in the presence of an N-methyl-D-aspartate receptor (NMDAR) antagonist. Interestingly, activation of the CREB/*c-Fos* system showed a relevant degree of colocalization with long-term synaptic plasticity. These results show that NMDAR-dependent plasticity at the cerebellum input stage bears about transcriptional and post-transcriptional processes potentially contributing to cerebellar learning and memory consolidation.

## Introduction

Long-term plasticity consists of changes in synaptic transmission and neuronal excitability (Hebb, 1949; Bliss and Lomo, 1973; Bienenstock et al., 1982; Bear and Abraham, 1996) that are thought to provide the basis for learning and memory in the brain (Bliss and Collingridge, 1993; Bliss et al., 2003; Bliss and Collingridge, 2013; Sweatt, 2016). Following induction, the expression of plasticity involves an early phase dominated by protein phosphorylation and a late phase (consolidation) requiring synthesis of new mRNA and proteins (Frey et al., 1988; Nguyen et al., 1994; Steward and Schuman, 2001; Steward and Worley, 2001; Pittenger et al., 2002; Barco et al., 2008; Santini et al., 2014). Consolidation often begins through an N-methyl-D-aspartate receptor (NMDAR)-mediated calcium increase promoting phosphorylation of the cAMP-responsive element binding protein (CREB), which is involved both in synaptic plasticity and in plasticity of intrinsic excitability (Flavell and Greenberg, 2008; Benito and Barco, 2010). CREB is a constitutive transcription factor (TF) that regulates the transcription of genes with a CRE site in their promoter leading to the expression of inducible TFs including the immediately early gene (IEG) *c-Fos* (Brindle and Montminy, 1992; West et al., 2002; Alberini, 2009). *c-Fos* transcription characterizes recently activated neurons and is needed for long-term potentiation (LTP; Morgan et al., 1987; Kaczmarek et al., 1988; Kaczmarek and Chaudhuri, 1997; Flavell and Greenberg, 2008), while *c-Fos* deletion impairs learning and memory (Fleischmann et al., 2003; Benito and Barco, 2015). The IEGs sustain the second wave of gene expression that includes effector genes regulating both the maintenance of plastic modifications and the establishment of homeostatic responses (Sheng et al., 1990; Nedivi et al., 1993; Qian et al., 1993; Benito and Barco, 2015).

Behavioral experiments in genetically modified mice suggest that the cerebellum, similar to the most studied hippocampus, could activate specific mechanisms for the consolidation of plasticity (Andreescu et al., 2011; Galliano et al., 2013; ten Brinke et al., 2015), and circumstantial evidence in cell culture suggests that the cellular substrate may reside in an NMDAR-dependent activation of the CREB/*c-Fos* system (Szekely et al., 1987, 1989; Bito et al., 1996; Deisseroth et al., 1998; Pons et al., 2001; Wu et al., 2001; Ciani et al., 2002; Monti et al., 2002; Bito and Takemoto-Kimura, 2003). However, whether the CREB/*c-Fos* system is activated in response to activity patterns inducing long-term plasticity of synaptic transmission and intrinsic excitability at the mossy fiber—granule cell (MF-GrC) relay (D’Angelo et al., 1999; Armano et al., 2000; Gall et al., 2005; Seja et al., 2012) is unknown. Puzzlingly enough, although GrCs show the highest NMDAR and CREB expression levels amongst all cerebellar neurons (Monaghan and Anderson, 1991; Brodie et al., 2004), the role of CREB in cerebellar synaptic plasticity has been so far investigated only for parallel fiber long-term depression (LTD) in Purkinje cells (PC; Ahn et al., 1999), which do not express postsynaptic NMDARs at these synapses (Piochon et al., 2010).

In the present investigation, we addressed the issue as to whether, in the cerebellar granular layer, long-term synaptic plasticity is accompanied by activation of the CREB/*c-Fos* system. By combining *in situ* hybridization and immunohistochemistry with voltage-sensitive dye imaging (VSDi; Gandolfi et al., 2015), our results reveal a relevant degree of colocalization of long-term synaptic plasticity with activation of the CREB/*c-Fos* system through an NMDAR-dependent mechanism. This activation of gene-expression and protein synthesis provides the basis for plasticity consolidation in the cerebellum granular layer.

## Materials and Methods

The experiments reported in this article were conducted on 18–25 day-old (day of birth is day 0) Wistar rats, according to the international guidelines from the European Union Directive 2010/63/EU on the ethical use of animals and approved by the local ethical committee of the University of Pavia, Pavia, Italy.

### Brain Slice Preparation

Acute cerebellar slices were obtained as previously reported (D’Angelo et al., 1999; Nieus et al., 2014). Briefly, rats were anesthetized with halothane (Sigma; 0.5 ml in 2 dm^3^ for 1–2 min) before being killed by decapitation. The cerebellum was gently removed, the vermis was isolated, fixed on a plastic support with cyano-acrilic glue, and immersed into a cold (2–3°C) cutting solution. Slices (220 μm thick) were cut in the sagittal plane with a vibroslicer (LEICA VT1200S). The cutting solution contained (in mM): K-gluconate 130, KCl 15, EGTA 0.2, Hepes 20, and glucose 10 (pH 7.4 with KOH). Slices were incubated for about 1 h before recordings at 31°C in oxygenated Krebs solution containing (in mM): NaCl 120, KCl 2, MgSO_4_ 1.2, NaHCO_3_ 26, KH2PO_4_ 1.2, CaCl_2_ 2, glucose 11 (pH 7.4 when equilibrated with 95% O_2_–5% CO_2_). For optical recordings, slices were pre-incubated for 30 min in oxygenated Krebs solution for VSD staining (see below). When needed, the extracellular solution was supplemented with the NMDAR blocker, 50 μM D-2-amino-5-phosphonovaleric acid (D-APV or simply APV; Tocris Cookson). Slices were gently positioned in the recording chamber and were immobilized with a nylon mesh attached to a platinum omega wire to improve tissue adhesion and mechanical stability. Perfusion of standard extracellular solution (2–3 ml/min) maintained at 32°C with a feedback temperature controller (Thermostat HC2, Multi Channel Systems, Reutlingen, Germany) was performed during the recording session.

### Electrical Stimulations, Plasticity Induction and Slice Processing

All experiments were conducted on lobules V, VI and VII. No difference in synaptic plasticity properties and underlying mechanisms have been observed in these lobules (e.g., D’Angelo et al., 1999; Maffei et al., 2003; D’Errico et al., 2009; Sgritta et al., 2017). The MFs were stimulated with square voltage pulses (±4–8 V; 200 μs) delivered individually. Voltage pulses were applied through a bipolar tungsten electrode connected to a pulse generator through a stimulus isolation unit. Synaptic plasticity was induced by eight bursts of 10 pulses at 100 Hz repeated every 250 ms (Theta Burst Stimulation, TBS). In a set of slices the TBS was not delivered. These slices were used as controls compared to those in which long-term plasticity was induced.

After VSDi recordings, the cerebellar slices were removed from the recording chamber at either 15 or 120 min from the application of the TBS, fixed overnight in 4% paraformaldehyde (PAF) in Phosphate Buffer Saline (PBS) supplemented with 20% sucrose for cryoprotection, then extensively washed in PBS-20% sucrose at 4°C, embedded in OCT and frozen in liquid nitrogen. Serial cryostat sections (5 μm) were obtained from TBS and control samples, collected onto Poly-L-Lysine coated slides and processed for immunohistochemistry or *in situ* hybridization. VSDi was conducted on all the 44 slices used in this study (24 of which were processed for immunohistochemistry, and 20 for *in situ* hybridization).

### Voltage-Sensitive Dye Imaging (VSDi)

The stock solution for VSDi contained the dye Di-4-ANEPPS (Molecular Probes) dissolved and in a Krebs-based solution containing 50% ethanol (Sigma) and 5% Cremophor EL (a castor oil derivative; Sigma). Slices for optical recordings were incubated for 30 min in oxygenated Krebs solution added with 3% Di-4-ANEPPS stock solution and mixed with an equal volume of fetal bovine serum (Molecular Probes) to reach a final dye concentration of 2 mM (Gandolfi et al., 2015).

The recording chamber was installed on an upright epifluorescence microscope (Olympus BX51WI; Olympus, Japan), equipped with a 20× objective (XLUMPlanFl 0.95 NA, water immersion; Olympus, Japan). The light generated by a halogen lamp (10V150W LM150, Moritex, Tokyo, Japan) was controlled by an electronic shutter (Newport Corporation,Irvine, CA, USA) and then passed through an excitation filter (*λ* = 535 ± 20 nm), projected onto a dichroic mirror (*λ* = 565 nm) and reflected toward the objective lens to illuminate the specimen. Fluorescence generated by the tissue was transmitted through an absorption filter (*λ* > 580 nm) to the CMOS camera (MICAM Ultima, Scimedia, Brainvision, Tokyo, Japan). The whole imaging system was connected through an I/O interface (Brainvision) to a PC controlling illumination, stimulation and data acquisition. The final pixel size was 4.5 × 4.5 μm with 20× objective. Full-frame image acquisition was performed at 0.5 kHz. Data were acquired and displayed by Brainvision software and signals were analyzed using routines written in MATLAB (Mathworks). At the beginning of recordings, a calibration procedure was adopted to ensure homogeneity across experiments. The dynamic range of the CMOS camera was calibrated by measuring background fluorescence and setting the average light intensity in the absence of stimulation to 50% of the saturation level. The background fluorescence was sampled for 50 ms before triggering electrical stimulation and was used to measure the initial fluorescence intensity (*F*_0_). The relative fluorescence change (Δ*F/F*_0_) was then calculated for each time frame. The signal-to-noise ratio was improved by averaging 10 consecutive sweeps at the stimulus repetition frequency of 0.1 Hz.

### VSDi Data Analysis

The analysis of VSDi recordings was performed as reported previously (Prestori et al., 2013; Gandolfi et al., 2015). Briefly, an automatic custom-made procedure (MATLAB, Mathworks, Natick, MA, USA) detected the average fluorescence signal before the stimulus (*F*_0_) and the peak response after the stimulus (*F*) and yielded the relative fluorescence changes (Δ*F/F*_0_). Activation maps were generated by assigning to each pixel the corresponding Δ*F/F*_0_, which was filtered (3 × 3 spatial filter; BrainVision) and transformed in pseudocolors to improve graphical visualization. The intensity and sign of synaptic plasticity were estimated by calculating the difference between values before and after TBS. Two criteria were used to remove spurious signals and improve detection of plasticity. First, the pixels showing variations larger than ±1σ (1 standard deviation) from the control period average were considered unstable and were discarded. Then, only variations >10% and persisting until the end of recordings were considered as LTP or LTD.

### On the Origin of the VSDi Signal

In the granular layer, although NMDARs are mostly expressed by GrCs, they have also been revealed in cerebellar Golgi cells (GoC; Cesana et al., 2013). However, we note that there are about 500 GrCs every GoC (Eccles et al., 1967; Harvey and Napper, 1991) and, even considering the higher GoC than GrC surface (50:3; D’Angelo et al., 1999), less than 3.4% of the VSDi signal might originate from GoCs (see Mapelli and D’Angelo, 2007). The VSDi signal might have been also be contaminated by MF terminals, which also change their electrogenic response during LTP (Maffei et al., 2002). However, the density of GrC somata is one order of magnitude higher than that of the glomeruli, where MF terminals reside (4 × 10^6^/mm^3^ vs. 3 × 10^5^/mm^3^; Jakab and Hámori, 1988), so that the postsynaptic surface generating the VSDi signal is about 40 times larger than the presynaptic surface (Gandolfi et al., 2015). Finally, while synaptic activation evokes responses in astrocytes, these have kinetics that are much slower (tens or hundreds of milliseconds) than those in neurons (a few milliseconds in GrC recordings, D’Angelo et al., 1995), so that it is unlikely that glial cells contributed to generating the VSDi signals. Therefore, most of the VSDi signal changes had to occur in GrCs, although a minor contribution coming from presynaptic MF terminals, glial cells, or other neuronal subtypes (e.g., GoCs) cannot be ruled out. The depth at which the VSDi signal was generated was annotated and the corresponding serial cryostat sections were considered for immunofluorescence and *in situ* hybridization processing.

### *In Situ* Hybridization

*In situ* hybridization was carried out as previously described (Laforenza et al., 2009) with minor modifications. Plasmids containing c-Fos probe corresponding to the fourth exon of the mouse *c-Fos* gene and *Creb* mouse mRNA were kindly provided by Fabio Tascedda Lab (Modena and Reggio Emilia University, Dept. of Life Sciences). Derived probes show respectively 100% and 97% homology with *Rattus norvegicus* corresponding mRNA sequences. Briefly, sections were air dried for 1 h at room temperature, post fixed 20 min in 4% PAF-PBS and treated 8 min at room temperature with Proteinase K (PK, 20 μg/ml in PBS). PK digestion was blocked washing with 0.2% glycin in PBS followed by a post fixation step of 20 min at room temperature. Sections were then dehydrated, delipidated by chloroform treatment and air dried. Prehybridization was carried out for 2 h at 52°C in hybridization buffer (50% deionized Formamide, 0.3 M NaCl, 20 mM Tris HCl pH 7.4, 5 mM EDTA, 10 mM NaH_2_PO_4_ pH 8.0, 10% dextran sulfate, 1× Denhardts Solution, 0.5 μg/ml Yeast RNA). Hybridization was carried out for 18–22 h at 48–52°C depending on probe in hybridization buffer containing 1 ng/μl of Digoxigenin (DIG) labeled antisense *c-Fos* or *Creb* riboprobe. After high stringency washes (to 0.2 × SSC at 60°C), sections were washed with PBS containing 0.1% tween 20 (PBS-T), blocked 1 h at room temperature with 2% blocking reagent (Roche) 10% sheep serum in PBS-T and incubated overnight at 4°C with a polyclonal sheep anti-DIG antibody conjugated to Alkaline phosphatase (Boehringer, 1:1000 dilution). Enzyme activity was visualized by 2–18 h reaction with NBT/BCIP chromogenic substrate (Boehringer) at 4°C in the dark. The sections were observed with a Nikon Eclipse 80i light microscope and images were acquired with the Nikon Nis Element F Imaging Software. *c-Fos* and *Creb* sense probes, as well as myosin antisense probe were used on parallel sections in each experiment as negative controls. All experiments were replicated on at least five independent samples for each experimental condition (control samples, 15 and 120 min TBS samples, APV samples) on all sections derived from each samples, giving consistent results.

### Immunohistochemistry

Double immunofluorescent labeling was performed as follows: (1) sections were dried 30 min at room temperature and washed with Tris-buffered saline; (2) sections were blocked 1 h in Tris-buffered saline containing 10% normal horse serum (NHS) and 0.3% Triton X-100 (TX-100) at room temperature; (3) sections were incubated overnight at 4°C in Tris-buffered saline/1% NHS/0.3% TX-100 containing a mixture of a goat anti-c-FOS antibody (sc-52, diluted 1:400; Santa Cruz) and a rabbit anti-phospho-CREB (P-CREB, Ser133) antibody (87G3, diluted 1:100, Cell Signaling); (4) sections were rinsed in Tris-buffered saline and incubated 1 h at room temperature in Tris-buffered saline /1% NHS/0.3% TX-100 containing a mixture of Alexa Fluor 488 conjugated donkey anti-goat IgG antibody (1:300) and Alexa Fluor 594 conjugated donkey anti-rabbit IgG antibody (1:300, Life Technologies); and (5) sections were rinsed in Tris-buffered saline and covered with Prolong with DAPI. Negative immunofluorescent controls were performed by omitting the primary antibody from the otherwise regular immunolabeling protocol.

### Immunofluorescence Image Acquisition and Analysis

Immunofluorescence images were acquired with a Zeiss Apotome microscope with a 20× magnification. The gain and offset levels of the CCD camera (Axiocam MR, Zeiss) and the intensity of the halogen lamp were kept constant throughout acquisitions. Initially, a wide-field reconstruction of the slice under investigation was obtained using AxioVision software 4.8.2 (Zeiss) in order to identify and select the stimulated lobule. Then, 20–30 images obtained with 20× magnification were combined through ICE software (Image Composite Editor, Microsoft) in order to preserve single cell resolution in wide-field reconstructions. The combined image was cropped in order to focus on the granular layer. It should be noted that the granular layer can be properly analyzed in parasagittal slices, whereas, on this section plane, Purkinje cell activation is incomplete due to interruption of parallel fibers in the molecular layer. The principles of image analysis can be summarized as follows. The intensity of fluorescence emission was analyzed using an RGB code, in which three different vectors are used to measure light intensity for the three fundamental colors (Red, Green, Blue). For each fluorophore, intensity matrices were generated by assigning to each pixel a value corresponding to a specific color in the RGB tensor. For instance, for a c-FOS image the intensity matrix was generated by assigning to all pixels the values corresponding to green in the RGB data set. Then, in order to better identify the colocalization of gene expression products (c-FOS, P-CREB) with cellular elements and to exclude non specific signals, the binding of DAPI to nucleic acids was exploited. The DAPI signal showed small variations (<0.05%; 12.2 ± 1.7 gray levels over 255; *n* = 12) and allowed therefore to clearly discriminate cellular structures. The RGB image was multiplied by the DAPI mask. In this way, the structures containing both nucleic acids and gene expression products were identified. Finally, for each pixel, the fluorescence *f*(*x*, *y*) was normalized with respect to average fluorescence ($\bar{f}$) yielding,

(1) $$F(x,y)\%=\frac{f(x,y)-\bar{f}}{\bar{f}}*100$$

*F(x, y)%* was used to generate correlation maps of c-FOS and P-CREB expression. In control slices, the average *F(x, y)%* was 4.3 ± 1.2% (*n* = 10).

### *In Situ* Hybridization Image Acquisition and Analysis

*In situ* hybridization images were acquired with a Nikon Eclipse 80i light microscope with a 10× objective to obtain a wide field image of the slice and with a 20× objective to achieve single cell resolution for gene expression analysis. The gain and offset levels of the CCD camera (Nikon Nis Element F Imaging Software) and the intensity of the halogen lamp were kept constant throughout acquisition. Pseudocolor representation and intensity matrices of *Creb* and *c-Fos* levels were generated through a custom-written algorithm developed in MATLAB (Mathworks, Natick, MA, USA). RGB images were converted into grayscale images and the area corresponding to the granular layer was isolated. Then the gene expression correlation map was generated as a normalized change with respect to the average granular layer mRNA level using the equivalent for Equation 1 applied to *in situ* hybridization signals. In control slices, the average normalized signal (as for *F(x, y*)%) was 4.2 ± 1.5% (*n* = 8).

### Cross Correlation Analysis between VSDi Plasticity Maps and Protein/mRNA Level Changes

Cross-correlation was performed between congruent 20× maps of VSDi and protein or mRNA expression images. The *normalized correlation coefficient (C_x, y_)* was calculated with the following equation:

(2) $$C_{x,y}=\frac{1}{n-1}\sum_{x,y}\frac{\left(f(x,y)-\bar{f})(t(x,y)-\bar{t}\right)}{\sigma_{f}\sigma_{t}}$$

where *f(x, y)* are pixel intensity values in protein or mRNA maps (as in Equation 1) and, by analogy, *t(x, y)* are pixel intensity values in VSDi plasticity maps, *n* is the number of pixels composing the maps, $\bar{f}$ and $\bar{t}$ are the average intensity values of each map, *σ_f_* and *σ_t_* are the standard deviations from the average intensity values. The −1 < *C_x,y_* < 1 values have been used to generate correlation maps. The *global correlation* between maps was quantified by calculating the number of pixels showing *C_x,y_* > 0.9 and normalizing by the number of pixels.

In the histograms of Figures 5, 6, correlated areas were calculated as the average percent area undergoing either LTP or LTD (in the plasticity maps) that *positively* correlated with corresponding protein/mRNA expression levels. It has to be noted that LTP positively correlates only with protein/mRNA expression levels above average, while LTD positively correlates only with protein/mRNA expression levels below average. Therefore, correlated areas do not represent the entire granular layer area, but are referred to the areas specifically showing LTP or LTD.

**Figure 1:**
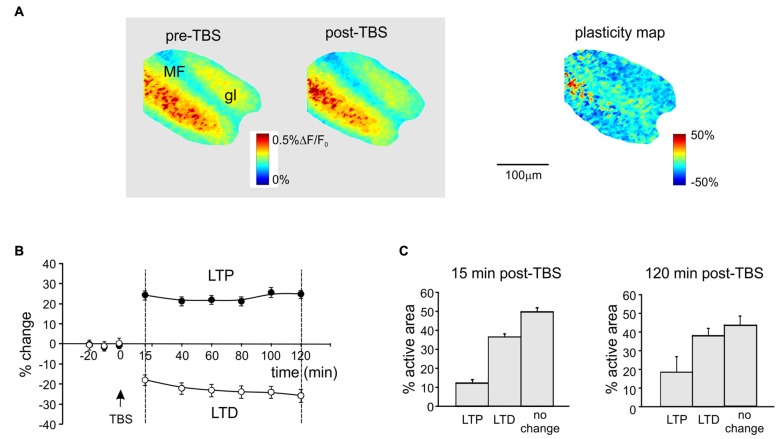
Spatial distribution of long-term potentiation and depression (LTP and LTD) in the granular layer after theta-burst stimulation (TBS). **(A)** Activation maps of the granular layer (elaborated from Voltage-sensitive dye imaging (VSDi) signals) in response to a single pulse delivered to the mossy fibers (MFs) before and 120 min after TBS (*left*). The plasticity map (*right*) reveals the distribution of changes induced by TBS. MFs, mossy fibers; gl, granular layer. **(B)** Average time course of LTP and LTD in eight different slices (mean and SEM, *n* = 8; see “Materials and Methods” Section for pixels acceptance criteria). The TBS is delivered at time 0. **(C)** Histograms showing the percentage of active pixels displaying LTP or LTD in the early phase (15 min, *left*) and in the late phase (120 min, *right*) of expression after TBS (mean and SEM, *n* = 8).

**Figure 2:**
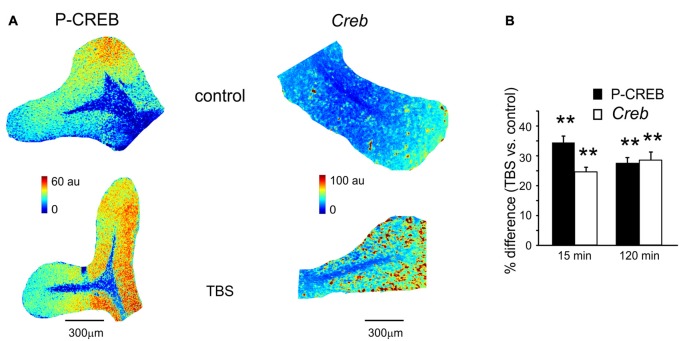
P-CREB and *Creb* levels in control and after TBS. **(A)** Pseudocolor maps of P-CREB and *Creb* levels measured in a control slice (*top*) and in a slice that has received TBS (*bottom*). Slices were fixed for immunofluorescence or *in situ* hybridization 15 min after TBS (or at an equivalent time in controls). **(B)** The histogram reveals differences of P-CREB and *Creb* levels between time-matched control slices and slices fixed either 15 min (mean and SEM, *n* = 10 for P-CREB and *n* = 8 for *Creb*) or 120 min after TBS (mean and SEM, *n* = 10 for P-CREB and *n* = 8 for *Creb*). Both P-CREB and *Creb* levels are significantly higher in slices that have received TBS than in control slices (**p < 0.01), both at 15 min and 120 min.

**Figure 3:**
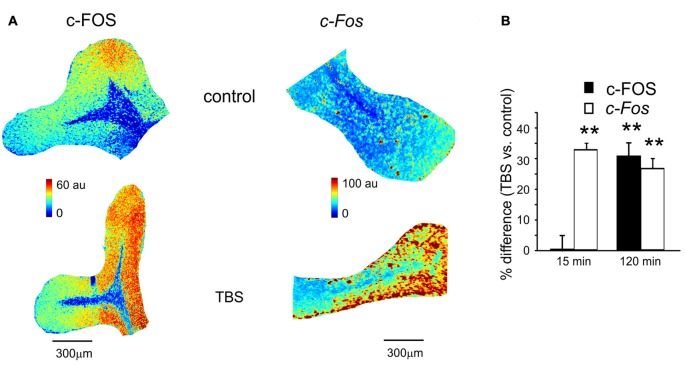
c-FOS and *c-Fos* levels in control and after TBS. **(A)** Pseudocolor maps of c-FOS and *c-Fos* levels measured in a control slice (*top*) and in a slice that has received TBS (*bottom*). Slices were fixed for immunofluorescence or *in situ* hybridization 120 min after TBS (or at an equivalent time in controls). **(B)** The histogram reveals differences of c-FOS and *c-Fos* levels between control slices and slices fixed either 15 min (mean and SEM, *n* = 10 for c-FOS and *n* = 8 for *c-Fos*) or 120 min after TBS (mean and SEM, *n* = 10 for c-FOS and *n* = 8 for *c-Fos*). Both c-FOS and *c-Fos* levels are significantly higher in slices that have received TBS than in control slices (**p < 0.01) at 120 min, but only *c-Fos* is increased at 15 min.

**Figure 4:**
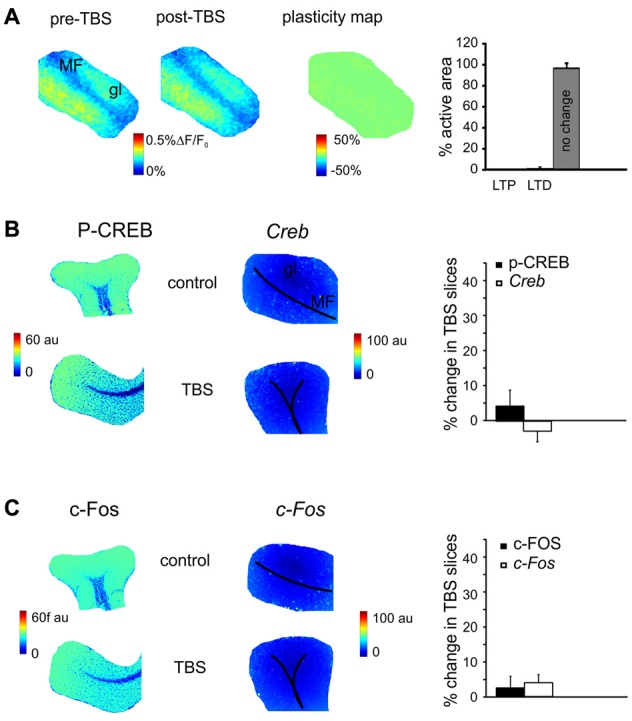
The effect of N-methyl-D-aspartate receptor (NMDAR) blockade on the long-term plasticity, gene expression and protein synthesis. **(A)** In the presence of 2-amino-5-phosphonovaleric acid (APV), the plasticity map (that was obtained as in Figure 1) shows that TBS does no longer induce synaptic plasticity. The histogram confirms the almost complete absence of changes in the slice. **(B)** In the presence of APV, the pseudocolor maps of P-CREB and *Creb* levels (that were obtained as in Figure 2) do not show remarkable differences between a control slice and a slice that has received TBS. Histograms show that no significant differences occur between control slices and slices that have received TBS (mean and SEM, *n* = 4; 120 min). **(C)** In the presence of APV, the pseudocolor maps of c-FOS and *c-Fos* levels (that were obtained as in Figure 2) do not show remarkable differences between a control slice and a slice that has received TBS. Histograms show that no significant differences occur between control slices and slices that have received TBS (mean and SEM, *n* = 4; 120 min).

**Figure 5:**
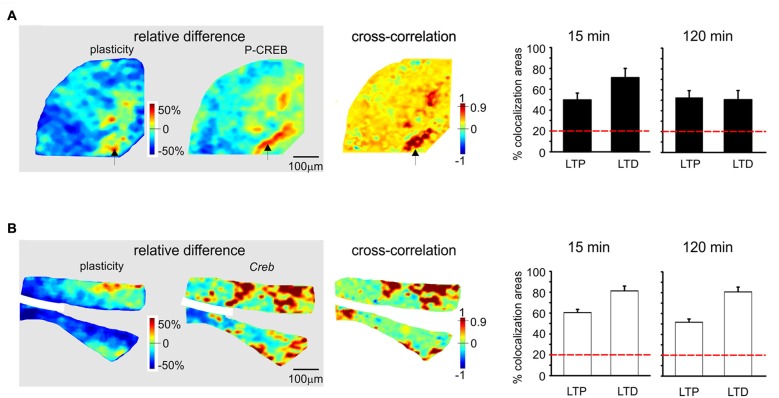
Spatial colocalization of LTP and LTD with the levels of activation of the CREB system following TBS. The maps on the left show the spatial distribution of LTP and LTD (i.e relative difference in circuit responsiveness with respect to controls) and protein/mRNA expression levels (as a relative difference with respect to the average expression level; Equation 1). The map on the right shows the cross-correlation between corresponding pixels in the other two maps (Equation 2). All the maps refer to measurements taken 120 min after TBS. The histograms show the percentage area showing colocalization of LTP and LTD with the relative levels of protein/mRNA at both 15 min and 120 min following TBS. The correlation is positive when LTP is associated with expression levels above average (both values are >0) and when LTD is associated with expression level below average (both values are <0). Only correlations with *C* > 0.9 have been used to construct the histograms (the values are normalized by the area making LTP or LTD). The dashed red lines represent the level of correlation between random matrices (i.e., the level at which correlation is due to noise). **(A)** P-CREB protein. The area showing colocalization of plasticity with P-CREB in these maps is 46.2% for LTP and 48.8% for LTD. **(B)** *Creb* mRNA. The area showing colocalization of plasticity with *Creb* in these maps is 43.1% for LTP and 66.6% for LTD.

**Figure 6:**
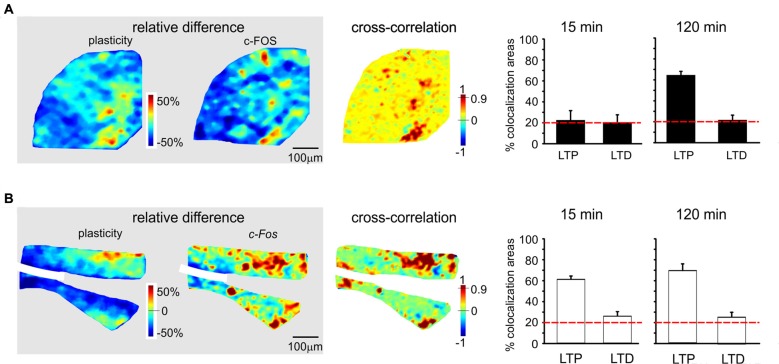
Spatial colocalization of LTP and LTD with the levels of activation of the c-FOS system following TBS. The maps on the left show the spatial distribution of LTP and LTD (i.e relative difference in circuit responsiveness with respect to controls) and protein/mRNA expression levels (as a relative difference with respect to the average expression level; Equation 1). The map on the right shows the cross-correlation between corresponding pixels in the other two maps (Equation 2). All the maps in this figure refer to measurements taken 120 min after TBS. The histograms show the percentage area showing colocalization of LTP and LTD with the relative levels of protein/mRNA at both 15 min and 120 min following TBS. The correlation is positive when LTP is associated with expression levels above average (both values are >0) and when LTD is associated with expression level below average (both values are <0). Only correlations with *C* > 0.9 have been used to construct the histograms (the values are normalized by the area making LTP or LTD). The dashed red lines represent the level of correlation between random matrices (i.e., the level at which correlation is due to noise). **(A)** c-FOS protein. The area showing colocalization of plasticity with c-FOS in these maps is 52.9% for LTP and 12.5% for LTD. **(B)** *c-Fos* mRNA. The area showing colocalization of plasticity with *c-Fos* in these maps is 53.9% for LTP and 33.3% for LTD.

### Statistics

Data are reported as mean ± standard error of the mean (SEM) and statistical comparisons were performed using Student’s *t*-test.

## Results

The delivery of theta burst stimulation (TBS, see “Materials and Methods” Section) to the MF bundle has been shown to induce long-term synaptic plasticity (Armano et al., 2000; Maffei et al., 2002; Sola et al., 2004; D’Angelo et al., 2005; Mapelli and D’Angelo, 2007; Prestori et al., 2013) at the MF-GrC relay. Here, we have combined VSDi (Gandolfi et al., 2015) with immunohistochemistry and *in situ* hybridization in order to investigate the potential involvement of the CREB/*c-Fos* system following TBS (Krug et al., 1984; Stanton and Sarvey, 1984; Frey et al., 2001; Lynch, 2004; Ahmed and Frey, 2005).

### Spatial Organization of LTP and LTD in the Granular Layer

Long-term synaptic plasticity was elicited by TBS and monitored using VSDi (Figure 1). The comparison of VSDi responses to MF stimulation before and after TBS allowed to generate plasticity maps (Figure 1A), which revealed the areas characterized by a persistent increase (LTP) or decrease (LTD) of neuronal activation. The changes in these areas followed typical LTP and LTD time-courses (Figure 1B), characterized by a rapid change that then persisted until 120 min after TBS. Between 15 min and 120 min after TBS, on average LTP was +29.3 ± 1.9% (*n* = 18 slices, *p* < 0.01) and LTD was −27.4 ± 2.1% (*n* = 18 slices, *p* < 0.01). The extension of the LTD area was larger than that of the LTP area both at 15 and 120 min after TBS (Figure 1C) confirming previous observations (Mapelli and D’Angelo, 2007; Gandolfi et al., 2015).

### CREB Activation and Creb Transcription during Plasticity

A first set of slices fixed at either 15 or 120 min after TBS was processed for immunohistochemical assessment of P-CREB, the active phosphorylated form of CREB (Figure 2A). The same procedure was applied to a set of slices that did not receive TBS (control slices, see “Materials and Methods” Section). In TBS slices compared to control slices, P-CREB levels increased already at 15 min (34.3 ± 3.8%; *n* = 10; *p* < 0.01) and remained high at 120 min after TBS (27.4 ± 2.7%, *n* = 10; *p* < 0.01; Figure 2B). Therefore, CREB phosphorylation started in an early phase after induction and was maintained during the late phase of plasticity.

A second set of slices fixed at either 15 or 120 min after TBS was processed for *in situ* hybridization (Figure 2A). The same procedure was applied to a set of slices that did not receive TBS (control slices, see “Materials and Methods” Section). Increased *Creb* mRNA levels were detected as soon as 15 min after TBS (24.2 ± 1.6% *n* = 8; *p* < 0.01) and remained high at 120 min (27.9 ± 2.8%, *n* = 8; *p* < 0.01; Figure 2B). Therefore, CREB phosphorylation and *Creb* gene transcription increase in the cerebellar granular layer soon after the induction of plasticity.

### c-FOS Expression and c-Fos Transcription during Plasticity

The same slices used for P-CREB and *Creb* mRNA assessment (as well as the corresponding control slices) were also used for c-FOS immunohistochemistry and *c-Fos in situ* hybridization (Figure 3A). Compared to time-matched control slices, c-FOS protein levels were not different 15 min after TBS (0.7 ± 2.4%, *p* < 0.01; *n* = 10) but were significantly higher at 120 min (30.6 ± 4.6%, *p* < 0.01; *n* = 10; Figure 3B). Conversely, *c-Fos* mRNA levels increased already 15 min after TBS (32.4 ± 2.6%, *p* < 0.01; *n* = 8) and remained high at 120 min (25.3 ± 2.9%, *p* < 0.01; *n* = 8; Figure 3B). These data are consistent with the non-constitutive nature of c-FOS expression, which would follow *c-Fos* transcription related to P-CREB (Fleischmann et al., 2003; Benito and Barco, 2015).

### The Effect of NMDARs Blockade on Plasticity and Activation of the P-CREB/c-Fos System

MF-GrC LTP and LTD are NMDAR-dependent (D’Angelo and Rossi, 1998; D’Angelo et al., 1999; Armano et al., 2000; Mapelli and D’Angelo, 2007). In order to determine whether the changes observed in gene expression and protein synthesis were also NMDAR-dependent, experiments were carried out in the presence of the NMDARs antagonist, APV (50 μM). In these experimental conditions, LTP and LTD were no longer detected in VSDi recordings (Figure 4A). At the same time, no significant differences were found either in the levels of P-CREB (3.5 ± 3.7%, *p* > 0.4; *n* = 4), c-FOS (2.8 ± 3.8%, *p* > 0.5; *n* = 4), *Creb* (−2.3 ± 4.6%, *p* > 0.3; *n* = 4) and *c-Fos* (4.5 ± 3.7%, *p* > 0.5; *n* = 4; Figures 4B,C). These observations indicate that protein synthesis and gene expression follow an NMDAR-dependent pathway like LTP and LTD in the cerebellar granular layer.

### Colocalization of Synaptic Plasticity and Activation of the CREB/c-Fos System

Since LTP and LTD on one side and the CREB/*c-Fos* system on the other were both dependent on synaptic NMDAR activation by TBS, we tested whether these different phenomena were spatially colocalized. To this aim, we correlated VSDi plasticity maps with the maps obtained in the same slices through immunohistochemistry and *in situ* hybridization (Figures 5, 6). It has to be noted that these maps reported expression values relative to average, therefore a positive correlation simply meant that e.g., LTP occurred where expression levels were above average and LTD where expression levels were below average.

P-CREB and *Creb* levels were colocalized above chance with both LTP and LTD, both in the early and late phase of plasticity (Figures 5A,B). Early phase: P-CREB (49.7 ± 6.6% LTP and 71.2 ± 8.6% LTD *n* = 5), *Creb* (60.5 ± 3.1% LTP and 81.2 ± 4.7% LTD *n* = 4). Late phase: P-CREB (52.7 ± 6.1% LTP and 50.6 ± 7.3% LTD *n* = 4), *Creb* (50.7 ± 6.3% LTP and 80.7 ± 6.1% LTD *n* = 4).

c-FOS levels showed a colocalization above chance only with LTP in the late phase (61.3 ± 3.7%, *n* = 4; Figures 6A,B). *c-Fos* mRNA showed a colocalization above chance only with LTP, both in the early phase (58.4 ± 4.9% LTP) and late phase (66.2 ± 5.2%, *n* = 4).

## Discussion

This article shows that, in the cerebellum granular layer, gene expression and protein synthesis can be initiated by the same MF activity patterns inducing long-term synaptic plasticity. The molecular pathway involved CREB phosphorylation followed by *Creb* and *c-Fos* transcription and c-FOS synthesis. Both LTP and LTD and the CREB/*c-Fos* system were NMDAR-dependent and showed a large degree of colocalization. These observations raise mechanistic hypotheses about the process of plasticity consolidation in the cerebellar network.

### Gene Expression and Protein Synthesis during Long-Term Synaptic Plasticity in the Cerebellar Granular Layer

The induction of long-term synaptic plasticity in the cerebellar granular layer was accompanied by the increase in CREB phosphorylation already 15 min after TBS. This close temporal association of CREB phosphorylation with the induction of synaptically-driven plasticity resembles that observed in other brain regions like hippocampus, amygdala, nucleus accumbens and locus coeruleus (Zhang and Linden, 2003; Dong et al., 2006; Han et al., 2006; Lopez de Armentia et al., 2007; Viosca et al., 2009; Benito and Barco, 2010). *Creb* and *c-Fos* mRNA were also increased already at 15 min after TBS, implying rapid initiation of transcription, while c-FOS protein levels increased later at 120 min, consistent with a delay required for new protein synthesis.

These observations conform to a general scheme (West et al., 2002; Alberini, 2009), in which P-CREB primes a *first wave* of TFs activation (Sheng et al., 1990; Nedivi et al., 1993; Qian et al., 1993; Benito and Barco, 2015), which is followed by a *second wave* involving the IEGs, *c-Fos* (Morgan et al., 1987; Kaczmarek et al., 1988; Brindle and Montminy, 1992; Kaczmarek and Chaudhuri, 1997; Fleischmann et al., 2003; Flavell and Greenberg, 2008; Jungenitz et al., 2014; Benito and Barco, 2015). A simpler scheme involving new protein synthesis using pre-existing mRNA (Otani et al., 1989; Huang and Kandel, 2005; Barco et al., 2008) may not apply to the present case.

The tight link between long-term synaptic plasticity, gene expression and protein synthesis is supported by the observation that all these processes were abolished by NMDARs blockade. NMDARs are highly expressed in GrCs and are necessary for the induction of long-term synaptic plasticity in the cerebellar granular layer (D’Angelo et al., 1999; Armano et al., 2000; Gall et al., 2005). In the molecular layer, similar mechanisms may be active on GrCs presynaptic terminals, where NMDARs play a key role in regulating parallel fiber—PC connection activity and plasticity (Bidoret et al., 2009; Bouvier et al., 2016). It is tempting to speculate that a common NMDAR-driven mechanisms could be responsible for the activation of a whole set of mechanisms leading to early expression and late consolidation of synaptic changes. In addition, NMDAR and CREB activation are involved in GrC development and survival (Ciani et al., 2002), suggesting a central role of the NMDAR- and CREB-dependent mechanisms for cerebellar network organization and function, as also reported for the hippocampus (Lonze and Ginty, 2002; Benito and Barco, 2010).

### Correlation of Gene Expression and Protein Synthesis with LTP and LTD

Before discussing the meaning of correlations between plasticity and the patterns of gene expression and protein synthesis (Figures 5, 6), it is useful to recall that, as for the definition given in “Materials and Methods” Section, the correlation is positive when LTP is associated with expression levels above average and when LTD is associated with expression levels below average.

The correlation between LTP and the P-CREB/*c-Fos* system is straightforward. Correlation analysis shows that LTP is accompanied by levels above average for all the elements of the P-CREB/*c-Fos* system. The regions generating LTP are the same that show P-CREB/*c-Fos* system activation above average. These spatial patterns are consistent with temporal patterns, in that LTP is correlated with P-CREB, *Creb* and *c-Fos* already at 15 min after induction, while LTP becomes correlated with c-FOS only at 120 min after induction.

Conversely, the correlation between LTD and the P-CREB/*c-Fos* system is more difficult to interpret. Indeed, a strong spatial correlation with LTD is found for P-CREB and *Creb* but not for c-FOS and *c-Fos*. It should also be noted that the correlation of P-CREB and *Creb* with LTD corresponds by definition to an expression level below average (rather than above average, as it is the case of LTP). Therefore, it is impossible to determine whether, during LTD, P-CREB and *Creb* increase less than in LTP, do not increase at all or even decrease below the basal level. The matter of fact is that there is a change in P-CREB and *Creb* in the areas showing LTD, for which we cannot provide a precise explanation.

As a whole, this correlation analysis strongly supports a colocalization of LTP with the activation of the P-CREB/*c-Fos* system. Moreover, it suggests that LTD, while involving P-CREB and *Creb* changes, might then proceed along consolidation mechanisms different from those of LTP, in agreement with observations reported in the hippocampus (Barco et al., 2002; Sajikumar et al., 2005; Young et al., 2006; Sajikumar et al., 2007; Barco et al., 2008).

### Comparison with Gene Expression in Neocortex and Hippocampus

These results suggest that the molecular mechanisms engaged in plasticity consolidation in the cerebellum granular layer bear similarities to those of more studied brain regions like the hippocampus and neocortex (Krug et al., 1984; Stanton and Sarvey, 1984; Frey et al., 2001; Karachot et al., 2001; Lynch, 2004; Ahmed and Frey, 2005). In particular, CREB phosphorylation in the hippocampus (e.g., Bito et al., 1996; Deisseroth et al., 1996; Benito et al., 2011) has been proposed to trigger different effectors (including second wave TFs) responsible for the structural (e.g., out-growth or remodeling of new spines) and functional changes characterizing long-term plastic changes. These effectors downstream to P-CREB include neurotrophins, molecules mediating cell adhesion and synaptic tagging, as well as mechanisms controlling neuronal intrinsic excitability and synaptic responsiveness, e.g., modified expression of glutamate receptors. Consistently, genetic manipulation of the P-CREB cascade was shown to alter learning and memory of behavioral tasks in rodents (Cole and Josselyn, 2008; Benito and Barco, 2010). The possibility that a similar pattern of changes would follow activation of the P-CREB/*c-Fos* system in the cerebellum granular layer warrant future investigation.

## Conclusion

To the best of our knowledge, this is the first attempt to investigate the involvement of TFs and IEGs expression in long-term plasticity at the cerebellar input stage in response to MF stimulation patterns with physiological relevance (Chadderton et al., 2004; Rancz et al., 2007; Roggeri et al., 2008; Ramakrishnan et al., 2016). Previous works were carried out on GrCs in culture and made use of pharmacological stimulation (Szekely et al., 1987, 1989; Bito et al., 1996; Deisseroth et al., 1998; Pons et al., 2001; Wu et al., 2001; Ciani et al., 2002; Monti et al., 2002; Bito and Takemoto-Kimura, 2003). Here, the spatial and temporal correlation of LTP and LTD with NMDAR-dependent processes of protein phosphorylation, gene expression and protein synthesis, supports the existence of mechanisms capable of consolidating long-term synaptic plasticity. These findings are in agreement with recent reports on diffused activation of GrCs in learning behavioral tasks (Giovannucci et al., 2017; Wagner et al., 2017). Future models of cerebellar learning and functioning (e.g., see Garrido et al., 2013; Casellato et al., 2015; Mapelli et al., 2015) will have to take into account that specific MF activity patterns activate processes of gene expression and protein synthesis in the granular layer that might prelude to memory consolidation.

## Author Contributions

DG and JM performed imaging experiments and analyzed the data, MP performed *in situ* hybridization experiments, SC performed immunohistochemistry experiments. ST, M-TF-A and FB contributed to histological experiments and data processing. AB contributed to article editing, LM contributed to imaging experiments and elaborated the whole dataset and text, and EDA coordinated the whole experimental and analysis work and article preparation.

## Conflict of Interest Statement

The authors declare that the research was conducted in the absence of any commercial or financial relationships that could be construed as a potential conflict of interest.
